# Effects of Combined Interventions of Physical Activity and Diet in Childhood Cancer Survivors: A Systematic Review

**DOI:** 10.3390/children11070853

**Published:** 2024-07-13

**Authors:** Carlos Vasconcelos, Patrícia Sousa

**Affiliations:** 1 Higher School of Education of Viseu, Ci&DEI, Polytechnic Institute of Viseu, 3504-510 Viseu, Portugal; 2 Higher School of Education of Viseu, Polytechnic Institute of Viseu, 3504-510 Viseu, Portugal; psousa@esev.ipv.pt

**Keywords:** exercise, nutrition, pediatric oncology, survivorship

## Abstract

Physical activity and diet have complementary benefits for attenuating adverse health effects that childhood cancer survivors experience after the end of treatment. This review aimed to explore the impact of combined physical activity and diet interventions on the health status of childhood cancer survivors. A thorough review of the literature was carried out using the Cochrane Library, PubMed, Scielo, LILACS and Web of Science databases for studies published until June 2024. Studies concerning survivors of childhood cancer of any type engaged exclusively in combined diet/physical activity interventions were included in this review. Three manuscripts met the inclusion criteria. The results indicated that combined physical activity plus diet interventions showed beneficial impacts for the intestinal microbiome, percentage of calories from sweet foods, daily consumption of sugary beverages, reduction in sedentary activity, and mood (1 out of 1 study for each of the presented outcomes). Although more rigorous studies are needed, these findings showed promising results regarding the impact of interventions involving both physical activity and diet on childhood cancer survivors

## 1. Introduction

Childhood is considered the period between the chronological ages of three and eleven years old [1]. In this life stage period, cancer ranks as the second leading cause of death globally, with leukemia being the most prevalent form of childhood cancer (comprising 28% of all cases), followed by brain tumors, lymphomas, and solid malignancies like Wilms tumors and neuroblastoma [2,3].

Significant improvements in the diagnosis and treatment of childhood cancer have resulted in a high survivorship rate [4]. According to Allemani et al. [5], these advances in treatments and supportive care lead to a survival rate of 80%. This positive trend results in concerns regarding the recovery of these patients, as childhood cancer survivors have higher risks of premature mortality and serious morbidity compared with healthy individuals [6]. Childhood cancer survivors often face several health challenges stemming from the illness and its therapies, including nausea, fatigue, decreased aerobic and anaerobic fitness, reduced muscular strength, impaired neuromuscular coordination, diminished pulmonary and physical function, cardiovascular challenges, lowered quality of life, discomfort, muscle mass loss, and decreased stamina, among other manifestations [7]. Cardiovascular complications, such as valvular dysfunction. Chronic cardiomyopathy, peripheral vascular disease, arrhythmias, pericardial disease, early coronary artery diseases, and stroke represent the main contributors to morbidity and mortality among individuals with cancer [8].

Although medical treatments have a substantial impact on disease remission, lifestyle behaviors adopted after treatment contribute significantly to the prevention of chronic conditions and the promotion of overall health in this population [9]. The World Health Organization defines lifestyle as identifiable behavioral patterns influenced by individual characteristics, social interactions, socioeconomic factors, and environmental conditions [10]. Diet and physical activity are two crucial lifestyle factors for childhood cancer survivors [11]. Due to several barriers, childhood cancer survivors have difficulties achieving physical activity and diet guidelines [12]. Thus, there is an urgent need for exercise and diet interventions in this population.

In the last 10 years, several reviews have been performed regarding the influence of physical activity and diet interventions on the health status of childhood cancer survivors. However, most of them were focused exclusively on exercise interventions [13,14,15,16,17,18]. Regarding diet interventions performed in childhood cancer survivors, only one review was identified [19].

In what concerns the influence of physical activity interventions on various health outcomes in childhood cancer survivors, Rapti et al. [13] reported positive effects on muscle strength, fatigue, physical activity participation levels, psychosocial health, cardiorespiratory fitness, physical function, brain volume and structure, and bone mineral density, with few and non-serious adverse effects.

Morales et al. [14] observed that physical activity reduced waist circumference and waist-to-hip ratio, enhanced endothelial function, and improved levels of physical activity.

Shi et al. [15] demonstrated that exercise programs with supervision showed notable improvements in body mass index, fatigue, muscle strength, and levels of physical activity, although no statistical significance was found in the quality of life.

The majority of studies analyzed by Moberg et al. [16] indicated an increase in physical activity levels and a decrease in fatigue among childhood cancer survivors.

Wogksch et al. [17] revealed that survivors of childhood cancer engaging in exercise exhibited reduced risk of cardiovascular disease, enhanced indicators of cardiovascular health, and decreased risk of all-cause mortality compared to inactive survivors. Physically fit survivors also demonstrated improved neurocognitive function and a reduced risk of all-cause mortality compared to unfit survivors.

Mizrahi et al. [18] found that interventions did not increase physical activity levels but yielded positive outcomes for physical function and psychological well-being.

In what concerns the influence of diet interventions on several health outcomes in survivors of childhood cancer, in the review of Cohen et al. [19], due to the scarcity and heterogeneity of studies included, conclusions regarding the efficacy of diet interventions for survivors of childhood cancer cannot be drawn. Based on the literature review, no reviews were found regarding the effects of combined physical activity and diet interventions on childhood cancer survivors. Thus, the objective of the present review was to examine the effects of combined physical activity and dietary interventions on the health status of survivors of childhood cancer.

## 2. Materials and Methods

The authors utilized the PRISMA (Preferred Reporting Items for Systematic Reviews and Meta-Analyses) criteria to carry out this systematic review [20].

### 2.1. Search Strategy

An electronic search was conducted to perform this systematic review across multiple academic databases, such as Cochrane Library, Pubmed, Scielo, LILACS, and Web of Science, for articles since the establishment of each database until June 2024, without any restrictions on the date of publication. The following keywords were used: ((lifestyle [Title] OR exercise [Title] OR physical activity [Title] OR diet * [Title] OR food [Title] OR nutrition * [Title] OR eating [Title]) AND (cancer [Title] OR oncological [Title])) AND (child * [Title]).

### 2.2. Selection of the Studies

Two authors conducted a review of the search results and screened publications retrieved from databases following predefined steps. Initially, articles underwent screening based on information from their titles and abstracts. Subsequently, potentially relevant articles were retrieved for a full-text review to assess their eligibility for inclusion in the study. The inclusion criteria consisted of (1) interventional studies and (2) studies that exclusively comprised a combination of physical activity and diet for survivors of childhood cancer. Exclusion criteria were focused on (1) duplicate studies and (2) types of studies. Observational studies: reviews, meeting abstracts, editorials, letters and protocol studies were not considered for this review; (3) Lack of access to abstract/full text; (4) interventions carried out during cancer treatment; (5) Interventions that mix exercise and/or diet with psychosocial care.

### 2.3. Data Extraction

Each chosen manuscript was assessed by authors to retrieve details concerning: (1) study characteristics (authors, country, design); (2) participants’ characteristics (age, sex, sample size); (3) measured outcome(s); (4) intervention group; (5) comparator group; (6) main results.

### 2.4. Risk of Bias Assessment

The risk of bias of all articles included in this review was assessed independently by two authors using the Cochrane risk of bias tool (RoB 1.0) [21] for randomized controlled trials and the ROBINS-I tool [22] for non-randomized controlled trials. RoB 1.0 measures seven domains, namely, random sequence generation, allocation concealment, blinding of participants and researchers, blinding of outcome assessment, incomplete outcome data, selective reporting, and other bias, while ROBINS-I measures seven domains, namely, bias due to confounding, bias in selection of participants into the study, bias in classification of interventions, bias due to deviations from intended interventions, bias due to missing data, bias in measurements of outcomes, and bias in selection of the reported result. For each domain, results were presented on a scale of low, some concerns, and high for the RoB 1.0 and low, moderate, serious, and critical risk of bias for the ROBINS-I.

## 3. Results

### 3.1. Study Screening

**Figure 1:**
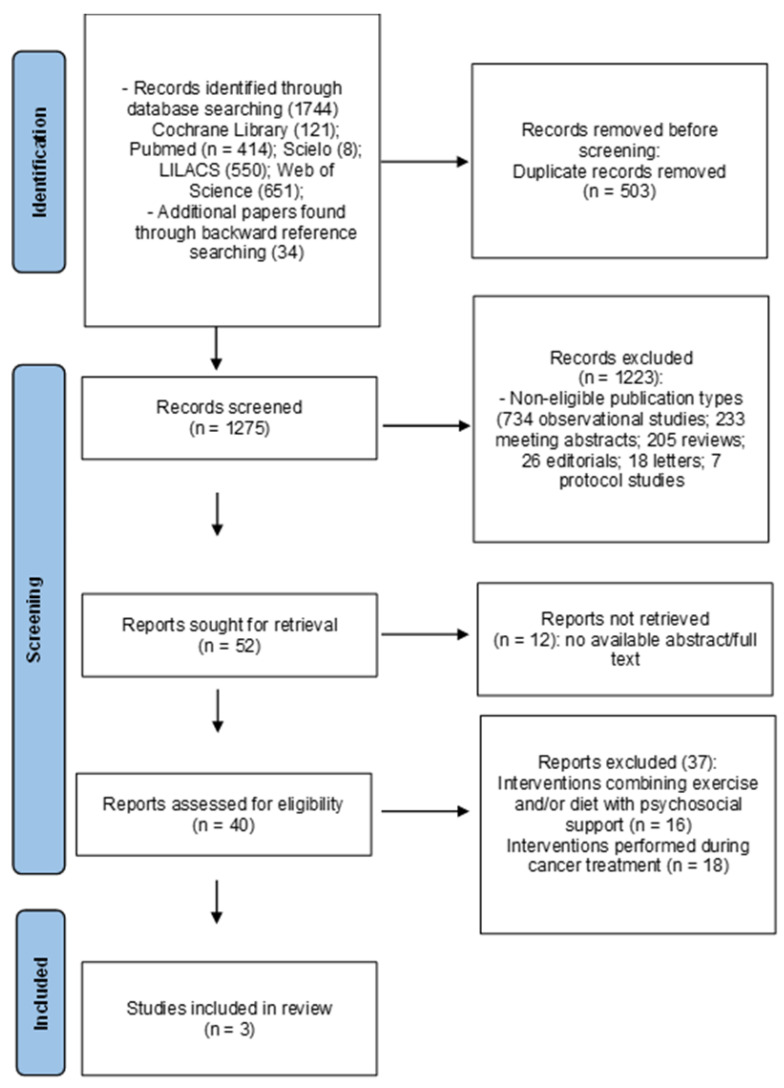
Flow diagram.

The initial search yielded 1778 references. After duplicate studies were removed (n = 503), 1275 manuscripts remained. Upon completion of the title and abstract screening, 1223 papers did not meet the inclusion criteria. Given that 12 references were not retrieved due to the unavailability of abstract/full texts, 40 studies were eligible for full-text review, of which 37 were excluded. Therefore, this systematic review contained a total of 3 studies (Figure 1).

### 3.2. Study Features

Of the three eligible studies [23,24,25], one was a randomized controlled trial (RCT) [25], and the other two were non-RCTs (one prospective cohort study [23] and a quasi-experimental study [24]). Two studies were carried out in the United States of America [24,25] and one study in Slovakia [23] between 2014 and 2023. The studies included in the review involved a total of 85 participants (39 females). The sample sizes ranged from 15 to 38, with participants’ mean/median age ranging from 8.9 to 14.8 years old.

### 3.3. Interventions

In the research conducted by Bielik et al. [23], an exercise program was composed of an individual online training program for 8 weeks, including 25–45 min of moderate to vigorous exercise, twice a week, under the supervision of a certified sports trainer. In terms of diet intervention, a probiotic dairy product (Danone, Belgium) has been provided once a day for 8 weeks. After the exercise and diet intervention, the authors identified a significant enhancement in: (1) the amount of Lactobacillus casei (*p* = 0.041); (2) the amount of the bacterial species Veillonella ratti (*p* = 0.022), and (3) the relative abundance of the bacterial species Veillonella rogosae (*p* = 0.003). The authors also observed an increase in the bacterial genera level of Veillonella, although this change was not statistically meaningful (*p* = 0.30).

According to the findings of Fuemeller et al. [24], physical activity plus diet intervention was performed through a smartphone app-based game for 8 weeks. Groups did not differ significantly on calorie consumption (*p* = 0.991); total fat (*p* = 0.686); dietary fiber (*p* = 0.332); servings of fruit and vegetables (*p* = 0.711); average minutes of moderate to strenuous exercise (*p*= 0.124); body mass index (*p* = 0.615); fruit and vegetable self-efficacy (*p* = 0.804) and physical activity self-efficacy (*p* = 0.515). On the contrary, significant differences between groups were found in the following variables: percentage of kcal from sweet foods (*p* = 0.049); average daily grams of sugary beverages (*p* = 0.038); percentage of time in sedentary activity (*p* = 0.038). It is, however, important to highlight that regarding the percentage of kcal from sweet foods, these significant results were attributed to a decrease in the consumption of these foods by the group that used the app <4 weeks and an increase in its consumption by the group that used the app >4 weeks.

As described by Wang et al. [25], subjects from the experimental group participated in a 4-month web, text, and phone counseling program for weight management. Participants from the control group were provided with printed materials on nutrition and physical activity for weight management. The dietary objectives for the intervention involved decreasing calorie consumption, tracking all foods and drinks consumed using a food log, controlling portion sizes, opting for foods and drinks with fewer calories rather than high-calorie alternatives, and making health-conscious food choices when dining out. The exercise objectives of the intervention aimed for at least one hour of moderate-to-strenuous exercise and aiming for 15,000 steps each day. For all participants, significant differences between groups were found on negative mood (*p* = 0.01), with improvements in the intervention group. Differences between groups in weight (*p* = 0.06), body mass index (*p* = 0.13), amount of physical activity (*p* = 0.65), energy intake (*p* = 0.24), interpersonal problems (*p* = 0.06), negative self-esteem (*p* = 0.07), and ineffectiveness (*p* = 0.91) were not statistically significant. For participants ≥ 14 years, at 4 months, the intervention group showed reduced weight gain (*p* = 0.05) and higher levels of moderate-to-strenuous exercise (*p* < 0.01). The remaining studied outcomes did not show statistically significant differences. Table 1 presents a summary of the articles included in this study.

### 3.4. Bias Risk Assessment

The risk of bias assessment for the included studies is presented in Table 2 for the randomized controlled trial and in Table 3 for the other studies. According to RoB 1.0, the investigation of Huang et al. [25] was rated as having a high risk of bias. Regarding the other studies, based on ROBINS-I, the research of Bielik et al. [23] has a moderate bias risk, while the research of Fuemeller et al. [24] has a serious risk. There was total agreement between the authors in this analysis.

## 4. Discussion

Health-promoting activities such as a healthy diet and regular physical activity might help mitigate certain risks linked to the long-term impact of treatment on childhood cancer survivors [26,27,28]. However, a high proportion of survivors of childhood cancer do not attain the dietary and exercise recommendations. [11]. According to Demark-Wahnefried [29], time constraints and fatigue comprised the primary barriers to exercise among cancer survivors. Besides the previous factors, Arroyave et al. [12] found another two constraints that hindered cancer survivors from performing physical activity: not belonging to a gym or preferring to do other activities (watch television, read a book, or spend time on a computer). In what concerns the barriers to the adoption of a healthier diet, time constraints, food availability at home and when dining out, taste, and willpower are common barriers among cancer survivors [11].

Dietary plus physical activity interventions allowed better results than dietary or physical activity programs alone, as shown in a systematic review conducted by Pérez-Bilbao et al. [29] with breast cancer patients. In this study [30], combined physical activity and dietary approaches led to significant improvements in muscle strength, cardiovascular fitness, body composition, fatigue, anxiety, depression, sleep quality, and quality of life compared to physical activity or diet interventions alone. In the present review, the three studies screened showed that dietary and physical activity interventions led to beneficial impacts on the intestinal microbiome [23], the percentage of kcal from sweet foods, the average daily grams of sugary beverages, a reduction in sedentary activity [24], and the mood [25] of childhood cancer survivors. However, none of the studies [23,24,25] make comparisons with a control group that participates in a physical activity or diet intervention.

In several other studies performed with childhood cancer survivors, health-promoting behaviors, including physical activity and diet, were also delivered using digital tools, such as active video games [31], text messaging [32], Facebook [33], websites [34,35]. Mobile technologies are increasingly being integrated into interventions targeting health behaviors [25]. These devices offer several advantages, including tailored information and messages sensitive to cultural nuances, swift access, interactive capabilities, and anonymity, which are particularly beneficial for addressing sensitive health issues. Leveraging the computational power and mobility of smartphones enables novel applications for delivering health messages in a timely and personalized manner. Since mobile phones are typically accessed by individuals, they enhance outreach, especially for delicate medical concerns [25]. The use of technology provides a viable, cost-effective alternative to in-person interventions, particularly suitable for reaching at-risk populations scattered across various locations [25]. Moreover, incorporating elements of gamification into promoting healthy lifestyle behaviors may hold particular appeal for this survivor group [24]. However, despite the increasing significance of technology, most adolescents still prefer face-to-face interactions over online delivery methods, as pointed out in the study of Touyz et al. [36]. In the same study, cancer survivors reported the local gym as the preferred location for the performance physical activity interventions. In other previous studies where interventions were exclusively of physical activity, they were conducted at hospitals [37,38,39,40,41,42], academic institutions [43], and home-based [44,45,46]. These interventions consisted of structured exercise programs, including aerobic exercise [42,47] or aerobic plus strength or flexibility exercise [39,41,43,48,49,50,51,52]. The duration of the exercise sessions was generally 30 to 45 min for aerobic exercise interventions and 45 to 60 min for interventions combining aerobic and strength training. The frequency of the exercise programs varied from twice daily to twice weekly, and the study durations ranged from six weeks to two years [39,41,42,43,47,48,49,50,51,52]. In the present review, the only study that was performed through a structured exercise program (although a distance-delivered intervention) [23] consisted of 25–45 min of aerobic plus strength training, twice a week for 8 weeks.

In what concerns diet interventions, we found two studies [53,54] that include face-to-face interaction mixed with remote nutrition education sessions.

Regarding parental participation in dietary and physical activity interventions in survivors of childhood cancer, a review by Raber et al. [55] reported interventions with no parental involvement [41,46,56,57,58,59,60,61], indirect parental involvement [25,39,43,48,49,50,51,52,62,63,64], or direct parental involvement [45,47,65]. In the studies with no parental involvement, parents were only referenced either for obtaining consent or to act as proxies to report behavioral data. Indirect approaches were employed to involve parents in the interventions, such as dispatching materials to their homes, extending invitations for joint sessions with their children, garnering parental support, and assigning tasks that needed a response (like parent satisfaction surveys) or action (such as actively observing home interventions). Fewer studies adopted direct methods to engage parents. In these studies [45,47,65], parents watched a 20-min explanatory video about the program and received identical study materials as their children for group discussions (facilitating mutual understanding and peer connection among parents). In the study of Mays et al. [45], parents contributed their insights in preliminary focus groups during program development. Furthermore, parents were directly encouraged to participate in activities exclusively for them, albeit not necessarily pertaining to specific parenting advice. Engagement with parents extended beyond surveys and proxy roles for their children, mandating the physical presence of one or both parents during exercise sessions, thus fostering familial involvement. [47,65].

## 5. Conclusions

This review showed that dietary plus physical activity interventions led to beneficial impacts on the intestinal microbiome, percentage of kcal from sweet foods, average daily grams of sugary beverages, reduction in sedentary activity, and mood (each outcome supported by 1 study).

Future studies related to dietary plus physical activity interventions on survivors of childhood cancer must: (1) involve childhood cancer survivors in the planning stages; (2) ensure interventions are tailored to their daily routines and interests; (3) early interventions, considering the early establishment of health behaviors; (4) repeat interventions and not only one intervention, as cancer survivors progress through various developmental stages; (5) incorporate combined approaches, including face-to-face interactions and web-based methods; (6) direct parental involvement.

Regarding research implications, future dietary and physical activity programs for childhood cancer survivors must prioritize larger sample sizes, as researchers can ensure greater statistical power and reliability in their findings, allowing for more robust conclusions. Additionally, a longer time of interventions and a longer period of follow-up will provide valuable insights into their long-term effectiveness and sustainability. Moreover, study populations must embrace diversity, including different ages, races, and ethnicities. This aspect will be crucial for the development of interventions that are really effective and equitable for all participants. Randomized controlled trials must also be prioritized, as this study design offers a methodological approach to reduce biases and enhance the validity of research findings, thereby facilitating more accurate assessments of intervention effectiveness. It will also be crucial to make a comparison with a control group that participates in a physical activity or diet intervention. To address challenges such as participant attrition, researchers must develop strategies to increase retention rates throughout the duration of studies. This may involve personalized approaches tailored to the specific needs and preferences of participants, as well as proactive measures to mitigate barriers to engagement. Furthermore, future research should explore the optimal content, duration, setting, mode of delivery, and timing of interventions to accommodate the diverse needs of various age cohorts and minority populations, including cancer survivors. Tailoring interventions to individual preferences and circumstances can maximize their efficacy and impact. Lastly, considering the heterogeneity of cancer types, future studies should investigate interventions tailored to the specific needs and challenges associated with different forms of cancer. Researchers can develop targeted interventions that optimize outcomes and enhance the well-being of cancer survivors across the spectrum of disease types.

## Figures and Tables

**Table 1:** Description of the studies.

Author/Country/Design	Participants’ Characteristics	Variables	Experimental Group	Control Group	Main Results
Bielik et al. [23]/Slovakia/Prospective study	Intervention group: 16 subjects (8.9 ± 3.3 years; 9 female) Control group: 16 subjects (9.0 ± 3.3 years; 9 female)	Gut microbiome	Individual online training program for 8 weeks included 25–45 min. of moderate to strenuous exercise (twice a week). The probiotic dairy product (Danone, Belgium) has been provided once a day for 8 weeks.	No intervention was precribed.	There were notable changes in bacterial diversity between pre- and post-intervention. Furthermore, it was found an increment in the amount of Lactobacillus casei and in some strains of Veillonella
Fuemmeler et al. [24]/USA/Quasi-experimental study	15 subjects (14.8 ± 2.0 years; 6 female).	- Energy intake - Total fat - Dietary fiber - Servings of FV - MVPA - BMI - FV self-efficacy - PA self-efficacy - Sweet foods - Sugary bevegares - Sedentary activity	Mobile app game designed to encourage healthy eating and physical activity during 8 weeks Intervention group was splited in two different groups regarding the use of the app: group 1 (8 individuals): >4 weeks of app use; group 2 (7 individuals): <4 weeks of app use)	Non applied for this study	No significant differences were found on energy intake, dietary fiber, total fat, servings of fruit and vegetables, average minutes of moderate-to-strenuous physical activity, body mass index, fruit and vegetables self-efficacy and physical activity self-efficacy. Significant differences were found on percent of kcl from sweet foods, daily average consumption of sugary drinks and proportion of time engaged in sedentary behaviors
Huang et al. [25]/USA/Randomized controlled trial	38 subjects (19 on experimental and 19 on control group) (Median = 13 years; 15 female)	- Negative mood - Weight - Body mass index - Amount of physical activity - Energy intake - Interpersonal problems - Negative self-esteem - Innefectiveness	Participated in a 4-month program combining web, text, and phone counseling for weight management.	Provided with printed materials on diet and physical activity for weight management.	Significant differences between groups were found on negative mood, with improvements in the intervention group Between groups, differences in weight, physical activity, body mass index, calorie intake, interpersonal problems, negative self-esteem and innefectiveness were not statistically significant

**Table 2:** Assessment of bias per RoB 1.0.

Criteria/Study	Huang et al. [25]
1. Random Sequence Generation	High
2. Allocation Concealment	High
3. Blinding of Participants and Personnel	High
4. Blinding of Outcome Assessment	High
5. Incomplete Outcome Data	Low
6. Selective Reporting	Some Concerns
7. Other Bias	High
Overall Risk of Bias	High

**Table 3:** Assessment of bias per ROBINS-I.

Criteria/Study	Bielik et al. [23]	Fuemmeler et al. [24]
1. Bias due to Confounding	Moderate	Moderate
2. Bias in Selection of Participants	Moderate	Serious
3. Bias in Classification of Interventions	Low	Low
4. Bias due to Deviations from Intended Interventions	Low	Low
5. Bias due to Missing Data	Low	Low
6. Bias in Measurement of Outcomes	Low	Low
7. Bias in the Selection of the Reported Result	Low	Low
Overall Risk of Bias	Moderate	Serious
